# Identification of common signature genes and pathways underlying the pathogenesis association between nonalcoholic fatty liver disease and atherosclerosis

**DOI:** 10.3389/fcvm.2023.1142296

**Published:** 2023-03-30

**Authors:** Shuangyang Mo, Yingwei Wang, Xin Yuan, Wenhong Wu, Huaying Zhao, Haixiao Wei, Haiyan Qin, Haixing Jiang, Shanyu Qin

**Affiliations:** ^1^ Gastroenterology Department, The First Affiliated Hospital of Guangxi Medical University, Nanning, China; ^2^ Gastroenterology Department, Liuzhou Peoples’ Hospital Affiliated to Guangxi Medical University, Liuzhou, China; ^3^ Cardiovascular Department, Liuzhou Peoples’ Hospital Affiliated to Guangxi Medical University, Liuzhou, China

**Keywords:** atherosclerosis, bioinformatics, machine learning, diagnosis model, immune infiltration, nonalcoholic fatty liver disease

## Abstract

**Background:**

Atherosclerosis (AS) is one of the leading causes of the cardio-cerebral vascular incident. The constantly emerging evidence indicates a close association between nonalcoholic fatty liver disease (NAFLD) and AS. However, the exact molecular mechanisms underlying the correlation between these two diseases remain unclear. This study proposed exploring the common signature genes, pathways, and immune cells among AS and NAFLD.

**Methods:**

The common differentially expressed genes (co-DEGs) with a consistent trend were identified *via* bioinformatic analyses of the Gene Expression Omnibus (GEO) datasets GSE28829 and GSE49541, respectively. Further, the Gene Ontology (GO) and Kyoto Encyclopedia of Genes and Genomes (KEGG) enrichment analyses were performed. We utilized machine learning algorithms of lasso and random forest (RF) to identify the common signature genes. Then the diagnostic nomogram models and receiver operator characteristic curve (ROC) analyses were constructed and validated with external verification datasets. The gene interaction network was established *via* the GeneMANIA database. Additionally, gene set enrichment analysis (GSEA), gene set variation analysis (GSVA), and immune infiltration analysis were performed to explore the co-regulated pathways and immune cells.

**Results:**

A total of 11 co-DEGs were identified. GO and KEGG analyses revealed that co-DEGs were mainly enriched in lipid catabolic process, calcium ion transport, and regulation of cytokine. Moreover, three common signature genes (PLCXD3, CCL19, and PKD2) were defined. Based on these genes, we constructed the efficiently predictable diagnostic models for advanced AS and NAFLD with the nomograms, evaluated with the ROC curves (AUC = 0.995 for advanced AS, 95% CI 0.971–1.0; AUC = 0.973 for advanced NAFLD, 95% CI 0.938–0.998). In addition, the AUC of the verification datasets had a similar trend. The NOD-like receptors (NLRs) signaling pathway might be the most crucial co-regulated pathway, and activated CD4 T cells and central memory CD4 T cells were significantly excessive infiltration in advanced NAFLD and AS.

**Conclusion:**

We identified three common signature genes (PLCXD3, CCL19, and PKD2), co-regulated pathways, and shared immune features of NAFLD and AS, which might provide novel insights into the molecular mechanism of NAFLD complicated with AS.

## Introduction

Nonalcoholic fatty liver disease (NAFLD) and atherosclerosis (AS) are both common chronic metabolic-related diseases worldwide, characterized by oxidative stress, inflammation damage, lipid peroxidation, and immune response (1–3). Evidence indicates that NAFLD is an independent risk factor for AS and cardiovascular disease (CVD) (1, 4). These two diseases share similar clinical features, such as dyslipidemia, insulin resistance, and abdominal obesity. CVD is the chief cause of death among NAFLD patients (5), but the risk of NAFLD for CVD is independent of metabolic syndrome (MS) (6). Chronic liver conditions, including NAFLD, also can promote the progression of hepatic cirrhosis and subsequently hepatocellular carcinoma (HCC) (7). These emphasize the vital need for efficacious treatment of AS in these NAFLD individuals.

AS is a remarkable chronic inflammatory disease of the artery vessels, marked by intimal plaque formation (8). The advanced AS plaques with thin or thick fibrous cap atheroma are more unstable and harmful than the early ones (pathological intimal thickening and intimal xanthoma) (9). The sudden rupture of advanced AS plaques can result in acute myocardial infarction (AMI) or stroke, leading to the worldwide cause of paralysis and death (10). It is essential to prevent the progression from early harmless AS plaques to rupture-prone ones. The excessive immune cell infiltration, such as lymphocytes, macrophages, mast cells, and dendritic cells, is extraordinarily predominant in advanced AS (11). Many microarray studies illustrate that inflammatory-related genes are highly activated in the advanced AS plaques (12). Additionally, various pro-inflammatory cytokines and chemokines enhance inflammatory response within AS plaques by inducing chemotaxis of immune cells, resulting in the promotion of advanced AS (13).

The spectrum stages of NAFLD are from nonalcoholic fatty liver (NAFL) to nonalcoholic steatohepatitis (NASH), advanced fibrosis, and progressive cirrhosis. The outcomes of steatosis and steatohepatitis are very different. The steatosis rarely progresses to liver fibrosis and is considered mild NAFLD (14). In contrast, around 20% of NAFLD patients will progress to NASH, and NASH can progress to cirrhosis in up to nearly 20% of patients (15). It is reported that NASH also can significantly promote the progression of HCC and lead to an increased risk for resultant liver-related morbidity and mortality (16). Hence, NASH is part of the spectrum of advanced NAFLD (14). Immune cells play an independent risk factor and trigger the origination of inflammatory-related cytokines and chemokines, leading to hepatocyte inflammatory damage and a fibrogenic response in NASH. Noteworthy, multitudinous harmful inflammatory sources, including intrahepatic inflammation, circulating inflammatory cells, excess chemokines, adipose tissue inflammation, and unbalanced intestinal flora microenvironment, have been confirmed as the probable inducement of advanced NAFLD (17).

Although NAFLD is defined as a critical risk factor and promotion for advanced AS, the exact common molecule mechanisms and pathways that trigger the progression of these two inflammatory diseases to the advanced ones remain unclear. The NOD-like receptors (NLRs) signaling pathway that includes intracellular NLRs family members, related cytokines, caspases, and nuclear factor kappa B (NF-KB) might be one of the most fundamental pathways (18). Activating NLRs generally leads to the enhanced downstream NF-KB signaling pathway and finally mediates cellular inflammatory response and apoptosis. It reported that the NF-kB activation, which is closely associated with atherogenesis, is also an essential regulator of intrahepatic inflammation in advanced NAFLD (19). NLRs protein 3 (NLRP3) inflammasome modulates the effector pro-inflammatory cytokines such as IL-1β and IL-18 to promote the origination of reactive oxide species (ROS), accelerating the progression from NAFL to the advanced stage (20). Moreover, NLRP3 inflammasome activation and IL-1β secretion enhance the AS progression by driving vascular inflammatory response (21).

NAFLD and AS have remarkably similar features in many aspects, complications, clinical prognosis, inflammatory response, cytokines, immune infiltration, and signaling pathways. Furthermore, it is necessary to identify the biomarkers involved in the progression of advanced NAFLD and AS due to the poor clinical prognosis. Nowadays, along with the advance of high-throughput sequencing and microarray technologies, exploring the interaction transcription characteristic may provide a novel insight into the common pathogenesis of advanced AS and NAFLD. Bioinformatics has performed a vital role in life science research, which was utilized to analyze the differentially expressed genes and predict the potential therapeutic targets in a particular disease. Therefore, in this research, we analyzed the gene expression dataset, downloaded from the Gene Expression Omnibus (GEO) database, with bioinformatic methods and machine learning algorithms to identify the common signature genes, pathways, and immune cells among advanced AS and NAFLD. Meanwhile, the predictive diagnostic nomogram models were established and evaluated for these two diseases.

## Materials and methods

### Data source of microarray

**Figure 1:**
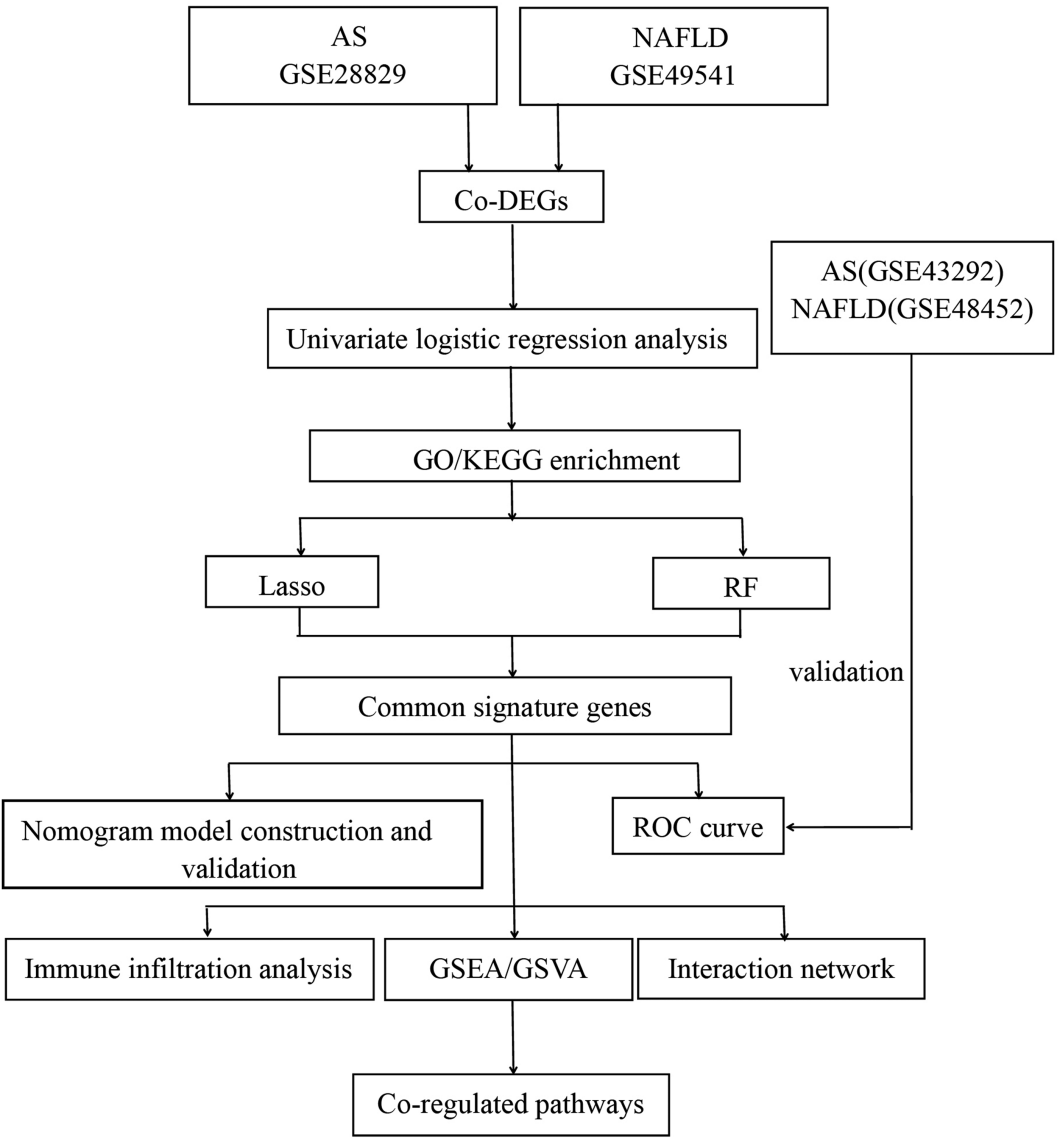
Flowchart of the study.

The mRNA expression profilings were downloaded from the public database of GEO. Atherosclerosis and nonalcoholic fatty liver disease were utilized as keywords to search for related gene expression datasets. The inclusion criteria were set as the test specimens included should be derived from humans, and these independent expression profiles contain the largest sample size. Four datasets (GSE28829, GSE49541, GSE43292, and GSE48452) were enrolled in this study. We divided them into a training set (GSE28829 and GSE49541) and a validation set (GSE43292 and GSE48452) because both GSE28829 and GSE49541 come from the same sequencing platform. There was 29 samples’ mRNA expression profiling in GSE28829, including 13 early (pathological intimal thickening and intimal xanthoma) and 16 advanced (thin or thick fibrous cap atheroma) atherosclerotic plaque samples from the Maastricht Pathology Tissue Collection (MPTC) (9, 22). In the dataset of GSE49541, a total of 72 NAFLD samples were divided into two groups based on the histologic severity of fibrosis: F0–1 (mild) and F3–4 (advanced) (14). Additional details are provided in Table 1, and the procedure for this study is displayed in Figure 1.

**Table 1:** Details of the GEO datasets.

Dataset	Disease	Platform	Organism	Number of samples
GSE28829	AS	GPL570	Homo sapiens	13 early atherosclerotic plaque samples 16 advanced atherosclerotic plaque samples
GSE49541	NAFLD	GPL570	Homo sapiens	40 mild NAFLD samples 32 advanced NAFLD samples
GSE43292	AS	GPL6244	Homo sapiens	32 control samples 32 atheroma plaque samples
GSE48452	NAFLD	GPL11532	Homo sapiens	14 NAFL samples 18 NASH samples

NAFLD, nonalcoholic fatty liver disease; NASH, nonalcoholic steatohepatitis; NAFL, nonalcoholic fatty liver; AS, atherosclerosis; GEO, Gene Expression Omnibus.

### Identification of co-DEGs

The differently expressed genes (DEGs) were identified from normalized and preprocessed data *via* the GEO2R tool (23). The screening threshold was stated at |log2 Fold Change (FC)| > 0.585 and *P* value < 0.05, and these DEGs with the consistent expression trends in GSE28829 and GSE49541 were picked up as co-DEGs. The correlation coefficients of co-DEGs were calculated based on Pearson's correlation coefficient.

### Enrichment analyses of GO and KEGG

Gene ontology (GO) enrichment [including biological process (BP), cellular component (CC), and molecular function (MF)] analysis and Kyoto Encyclopedia of Genes and Genomes (KEGG) pathway analysis were applied by utilizing the R clusterProfiler package. The false discovery rate (FDR) was calculated *via* the Benjamini-Hochberg adjustment. The cutoff criterion was *P*-value < 0.05. Finally, three packages (ggplot2, circlize, and pathview) were utilized to visualize these enrichment analyses’ significant results.

### Machine learning of lasso and random forest

Ulteriorly, two machine learning algorithms, containing lasso regression and random forest (RF), were used to screen the common signature genes from co-DEGs. Lasso regression and the optimal parameter *λ* were determined through 10-fold cross-validation *via* the R glmnet package with “family = binomial, measure = deviance” and with all other parameters arranged to default (24). In the RF algorithm, which comes with a feature selection function, the “Mean Decrease Gini” value could typify the significance of a feature. Each input gene of co-DEGs was ranked by order of importance in the classification using their “Mean Decrease Gini” score, and the top 50% of co-DEGs were identified as feature genes of the RF model. The particular co-DEGs identified by both machine learning models consistently were defined as the common signature genes.

### Construction of diagnostic model and evaluation of diagnostic efficiency

Using the R rms package, we constructed the diagnostic models with nomograms based on the common signature genes. The calibration curve was established to assess the calibration of the nomogram models by mean absolute error and 1,000 bootstrap samples using the R CalibrationCurves package. Decision curve analysis (DCA) was performed to evaluate the value of net benefits in the nomogram models at the different high-risk thresholds. Finally, whether the nomogram models had favorable predictive effects were evaluated by the clinical impact curve (CIC). Then the receiver operating characteristic (ROC) curves and the area under the curve (AUC) were applied to assess further the diagnostic efficacy of the nomogram models in the training set and validation set, respectively.

### Construction of interaction network for common signature genes

Subsequently, we established an interaction network of three common signature genes *via* GeneMANIA (http://www.genemania.org/), a reliable online tool for distinguishing internal correlations in gene sets. The details of the outcomes of this interaction network are displayed in Supplementary Material.

### Gene set variation analysis and gene set enrichment analysis

A nonparametric unsupervised gene set variation analysis (GSVA) method was performed to demonstrate the differential enrichment KEGG pathways in GSE28829 and GSE49541. This study utilized the R GSVA package with the gene sets of c2.cp.kegg.symbols.gmt, downloaded from the official site (https://www.gsea-msigdb.org/gsea/msigdb/). The threshold standard for statistically significant terms was set as adj. *P*-value < 0.05 and |log2FC|>1. Following, we focus on elucidating the potential roles of common signature genes in the advanced AS and NAFLD. A single-gene gene set enrichment analysis (GSEA) for each signature gene was performed separately *via* the R clusterProfiler package. Firstly, all samples from GSE28829 and GSE49541 were split into the low-expression and high-expression groups according to the expression level of a specific single signature gene. Then GSEA was played to estimate the significantly different KEGG pathways within these two groups.

### Immune infiltration analysis

Independent expression profiles GSE28829 and GSE49541 from the same sequencing platform were utilized to further immune infiltration analysis. The deconvolution algorithm of CIBERSORT (25), which can assess the percentage of 22 infiltrating immune cell subtypes, was used to calculate the immune infiltration of advanced AS plaques and advanced NAFLD tissues *via* the CIBERSORT R script v1.03. Then the correlation between each subtype of immune cells and each common signature gene was estimated with Pearson's correlation analysis and visualized. Furthermore, we obtained 28 immune-related cell gene sets and utilized the single sample gene set enrichment analysis (ssGSEA) *via* the R GSVA package to explore the different infiltration enrich scores of each immune cell subtype in each sample (26, 27). The R limma package was applied to analyze the different infiltration enrich scores between advanced and early AS groups, as well as advanced and mild NAFLD groups. Finally, the results of ssGSEA were visualized with the boxplots.

## Results

### Identification of co-DEGs in GSE28829 and GSE49541

**Figure 2:**
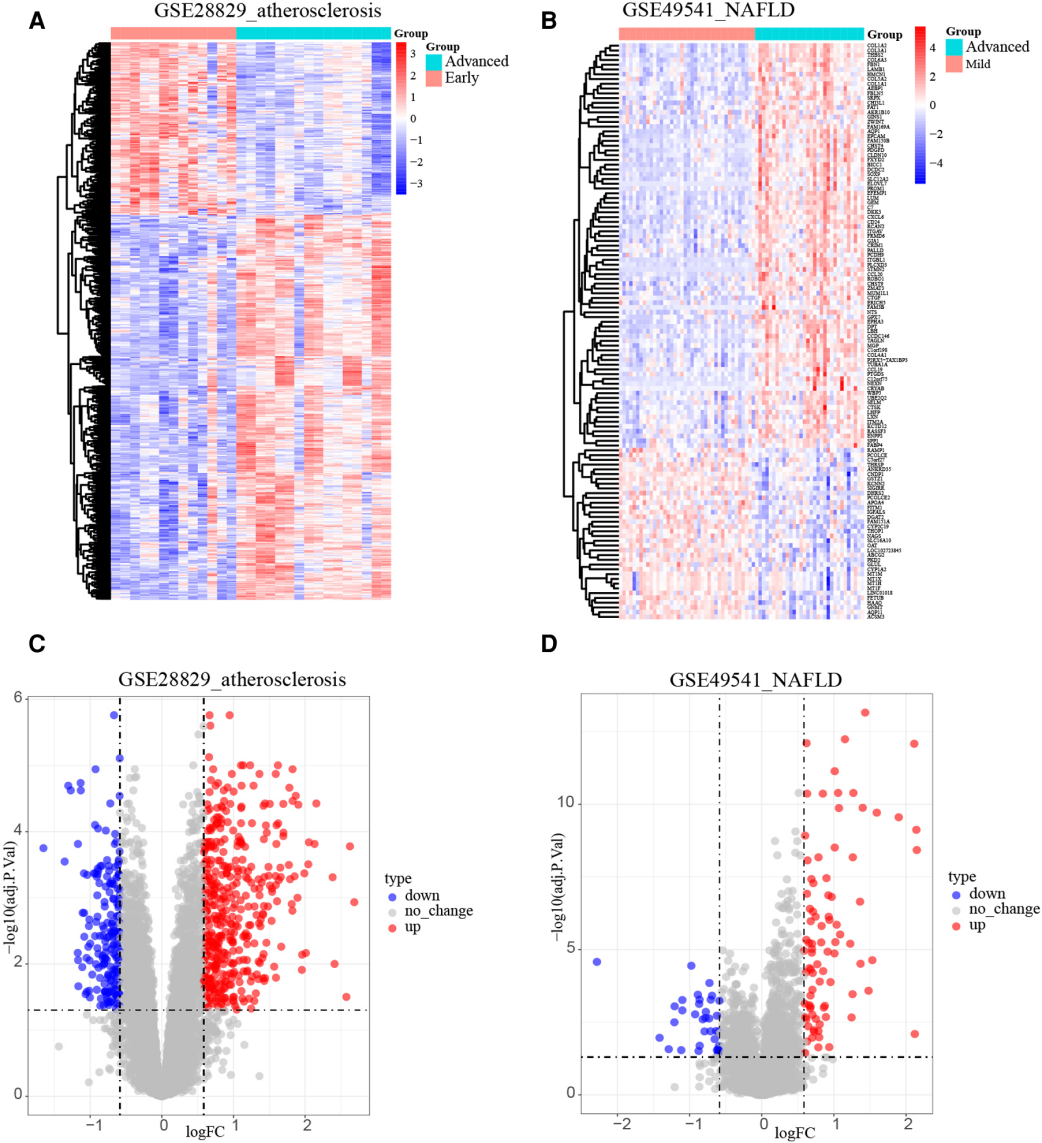
Identification of DEGs. (**A**) The heatmap of GSE28829; (**B**) the heatmap of GSE49541; (**C**) the volcano plot of GSE28829; (**D**) the volcano plot of GSE49541.

**Figure 3:**
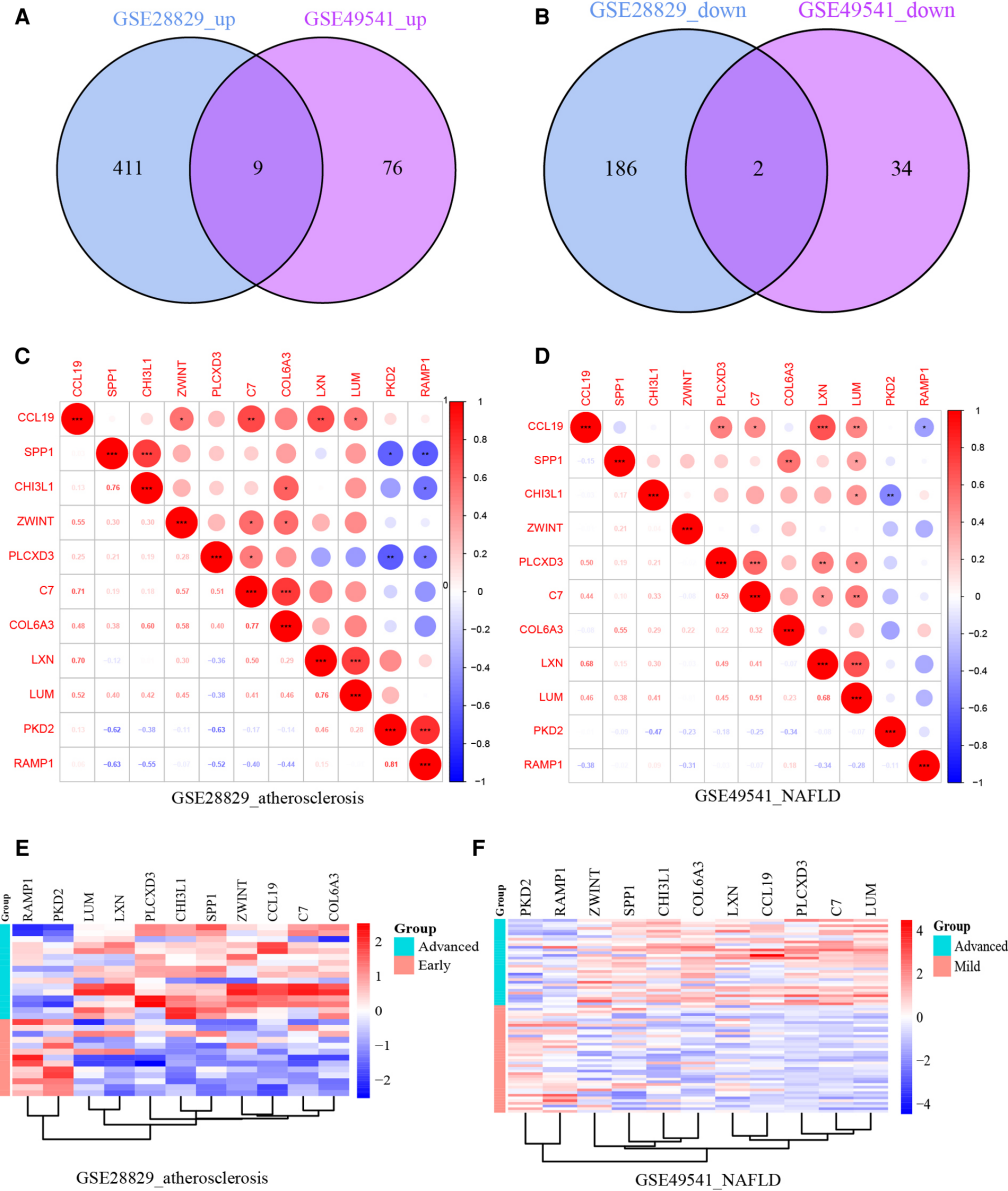
(**A**) The Venn diagram of 9 upregulated co-DEGs; (**B**) the Venn diagram of 2 downregulated co-DEGs; (**C**) the correlation coefficient matrix of co-DEGs in GSE28829; (**D**) the correlation coefficient matrix of co-DEGs in GSE49541; (**E**) the heatmap of co-DEGs in GSE28829; (**F**) the heatmap of co-DEGs in GSE49541. *means *P* < 0.05; **means *P* < 0.01; ***means *P* < 0.001.

After normalizing the micro-array results, DEGs (606 in GSE28829 and 121 in GSE49451) were identified with *P* value < 0.05 and |log2FC| > 0.585 as the screening threshold. Then the volcano plots and heatmaps of DEGs are displayed in Figures 2A–D. We applied the intersection of the Venn diagrams and identified 11 co-DEGs (9 upregulated co-DEGs and 2 downregulated co-DEGs) with consistent expression trends in GSE28829 and GSE49541 (Figures 3A,B and Supplementary Material). Then the heatmaps and correlation coefficient diagrams (Figures 3C–F) of these 11 co-DEGs show that it can easily identify patients with advanced AS or advanced NAFLD from early AS and mild NAFLD.

### Function enrichment analyses of the co-DEGs

**Figure 4:**
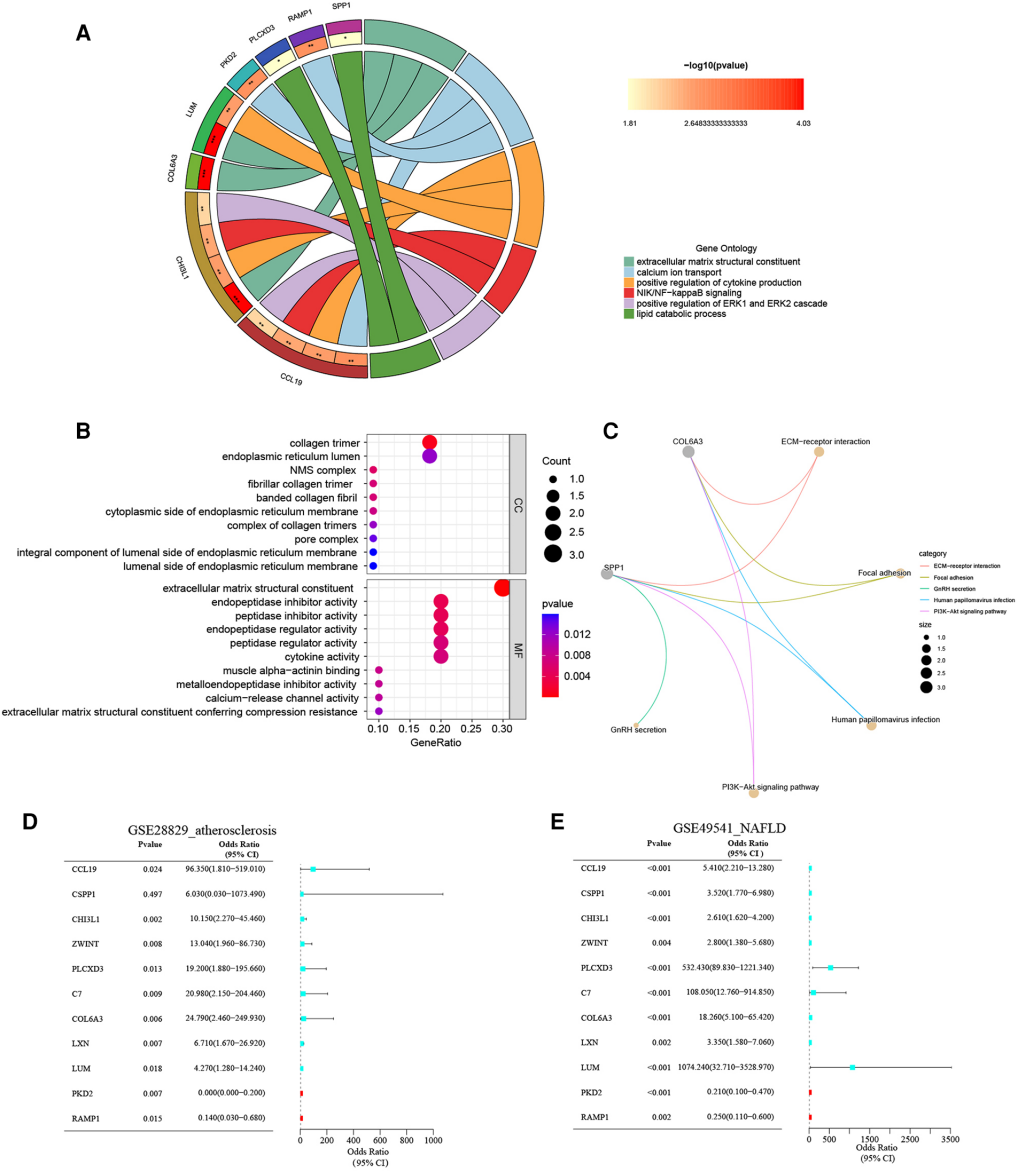
(**A**) The chord plot show GO BP enrichment significance items of co-DEGs; (**B**) the bubble chart show GO CC and GO MF enrichment significance items of co-DEGs; (**C**) the KEGG pathway enrichment analysis of co-DEGs; (**D**) forest map of univariate logistic regression of co-DEGs in GSE28829; (**E**) forest map of univariate logistic regression of co-DEGs in GSE49541.

The biology functions of co-DEGs were performed in GO and KEGG pathway analyses to gain more insights. In the GO category, these co-DEGs were clustered into three functional groups: BP, CC, and MF. The co-DEGs were mainly located in the reticulum and played a crucial role in extracellular matrix structural constituent, calcium ion transport, positive regulation of cytokine production, NIK/NF-KB signaling, positive regulation of ERK1 and ERK2 cascade, and lipid catabolic process. In the MF term, these co-DEGs participate in the regulation of extracellular matrix structural constituents, cytokine activity, muscle alpha-actinin binding, and calcium-release channel activity (Figures 4A,B and Supplementary Material). The enrichment analysis results of KEGG pathways show that the co-DEGs mostly associate with the ECM-receptor interaction, focal adhesion, and PI3K-Akt signaling pathway (Figure 4C and Supplementary Material). In summary, these outcomes powerfully demonstrate that cytokines, signal transduction pathways *via* calcium channels, and lipid metabolism cooperatively took part in these two inflammatory diseases.

### Univariate logistic regression analysis of the co-DEGs

The unvaried logistic regression analyses were utilized to reveal the correlation between each co-DEGs and dependent variables (advanced AS vs. early AS; advanced NAFLD vs. mild NAFLD). Expectedly, the upregulated co-DEGs were the risk factors for advanced AS and advanced NAFLD with the odds ratio (OR) > 1 simultaneously. On the contrary, the downregulated co-DEGs were protective factors. The forest plots for risk factors based on univariate analysis are shown in Figures 4D,E. These results suggest that these co-DEGs may be involved in the common pathogenesis of advanced AS and NAFLD.

### Recognition of common signature genes *via* machine learning algorithm

**Figure 5:**
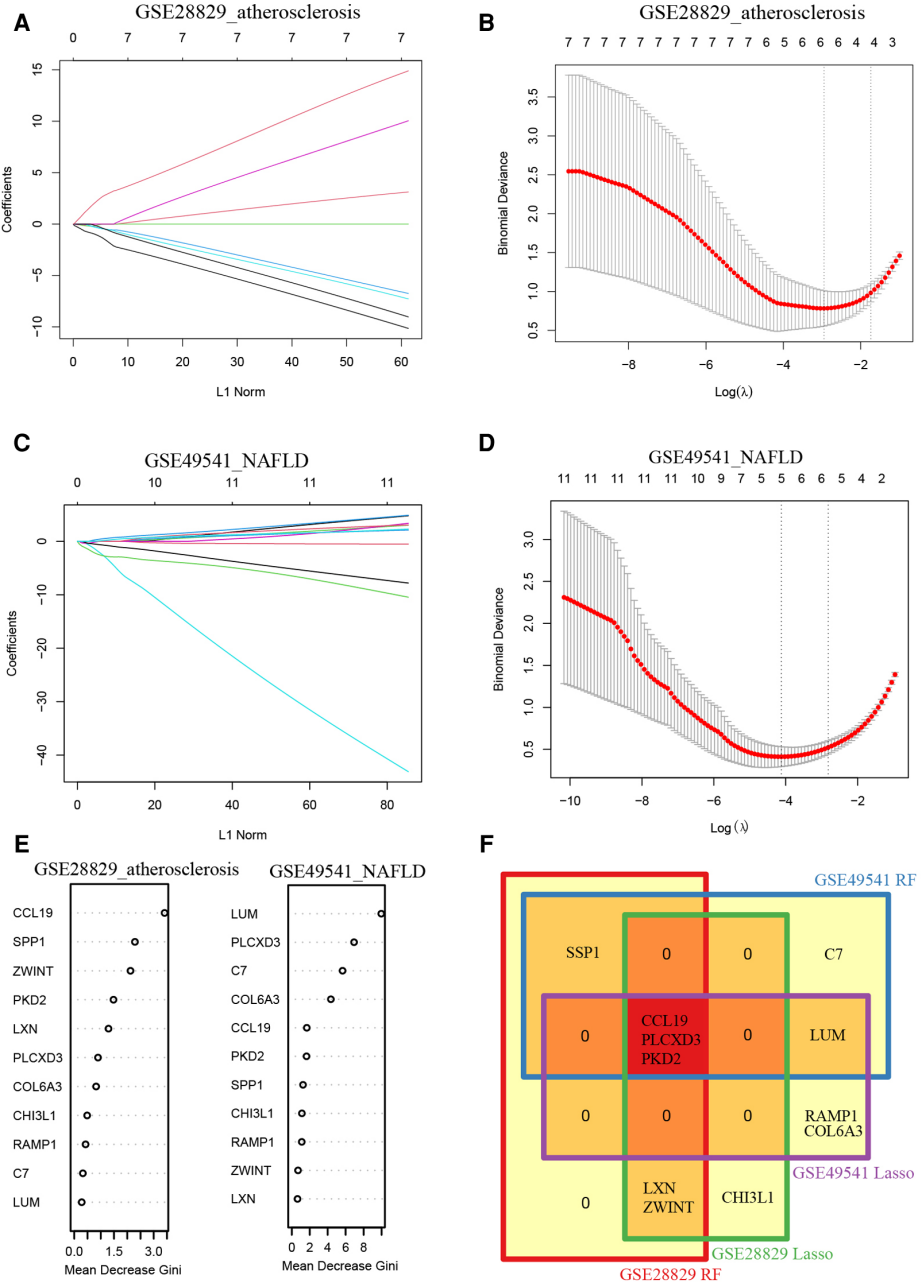
Recognition of common signature genes between AS and NAFLD. (**A**) The processes of LASSO regression for identifying variables in GSE28829 and mapping each variable to a curve; (**B**) the log (*λ*) value was optimally selected in GSE28829 by 10-fold cross-validation and plotted by the partial likelihood deviance; (**C**) the processes of LASSO regression for identifying variables in GSE49541 and mapping each variable to a curve; (**D**) the log (*λ*) value was optimally selected in GSE49541 by 10-fold cross-validation and plotted by the partial likelihood deviance; (**E**) the Mean Decrease Gini of each co-DEGs within random forest models for GSE28829 and GSE49541; (**F**) the Venn diagram of 3 common signature genes between GSE28829 and GSE49541.

Additionally, we trained machine learning algorithms of lasso regression and RF to screen the signature genes from co-DEGs. The lasso regression is a machine learning algorithm involving a linear relationship assumption and an L1 regularization penalty. Firstly, the lasso regression with the minimum binomial deviance was performed through 10-fold cross-validation. Then genes with non-zero regression coefficients were selected for signature genes of co-DEGs. As a result, there were 6 co-DEGs (CCL19, CHI3L1, ZWINT, PLCXD3, LXN, and PKD2) were included in the simplified lasso regularization model from GSE28829 (Figures 5A,B). Meanwhile, PLCXD3, COL6A3, LUM, PKD2, RAMP1, and CCL19 were enrolled from GSE49541 (Figures 5C,D). Then the importance of each co-DEGs was estimated by calculating the “Mean Decrease Gini”, and the top 6 co-DEGs were enrolled from the RF model in GSE28829 and GSE49541 (Figure 5E). Finally, an intersection of the Venn diagram was performed, and PLCXD3, CCL19, and PKD2 were simultaneously the signature genes for these two diseases (Figure 5F).

### Construction of the nomogram models

**Figure 6:**
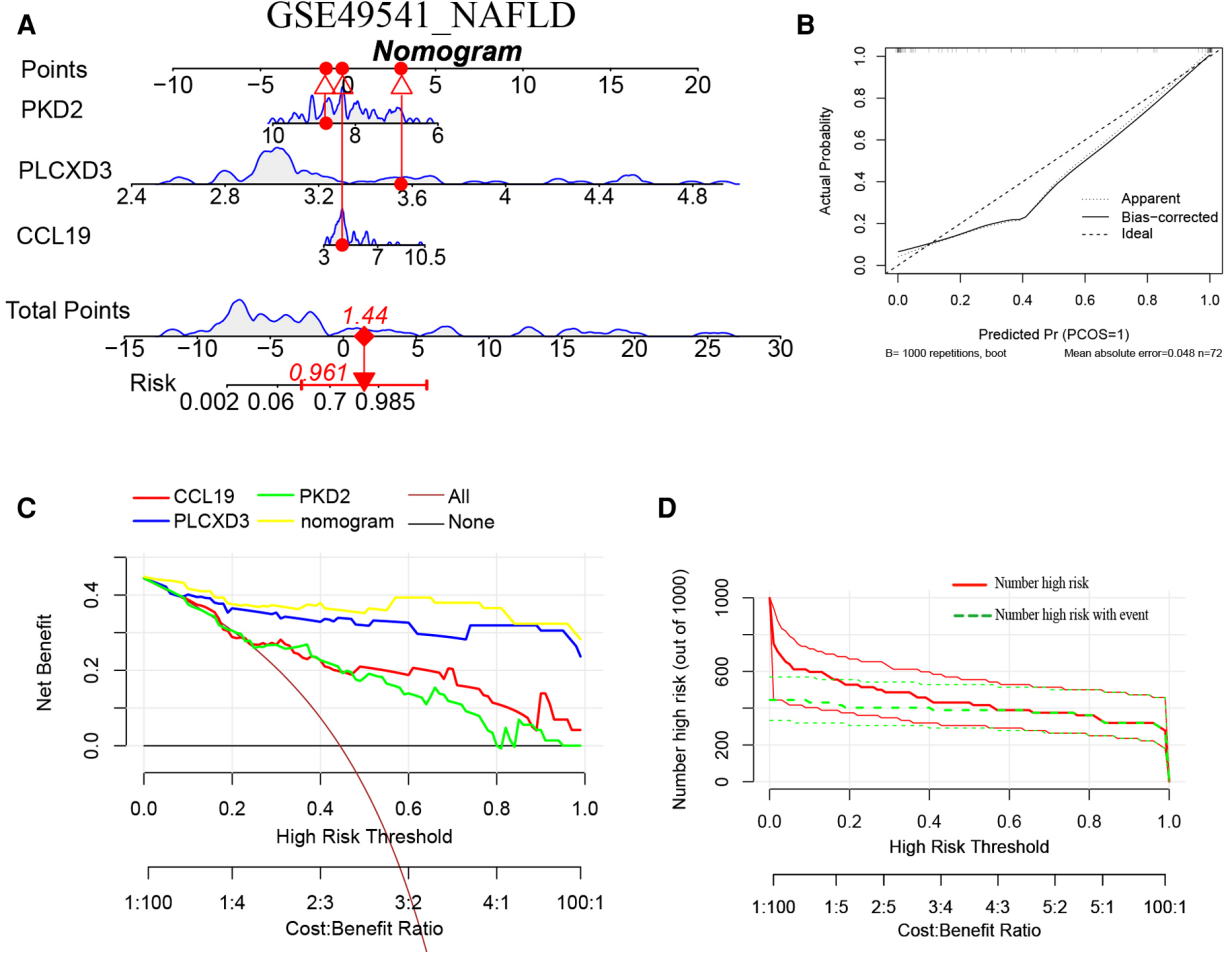
(**A**) The nomogram model predicting NAFLD based on three common signature genes in GSE49541. The nomogram is used by summing all points identified on the scale for each variable. The total points projected on the bottom scales indicate the probabilities of NAFLD; (**B**) the calibration curves for the nomogram with the mean absolute error = 0.048; (**C**) DCA of the nomogram model and each common signature gene (the “All” means diagnosis-all strategy; the “None” means diagnosis-none strategy) and the nomogram model had the highest net benefit at all practical risk thresholds (from 0 to 1.0); (**D**) the CIC of the nomogram model.

**Figure 7:**
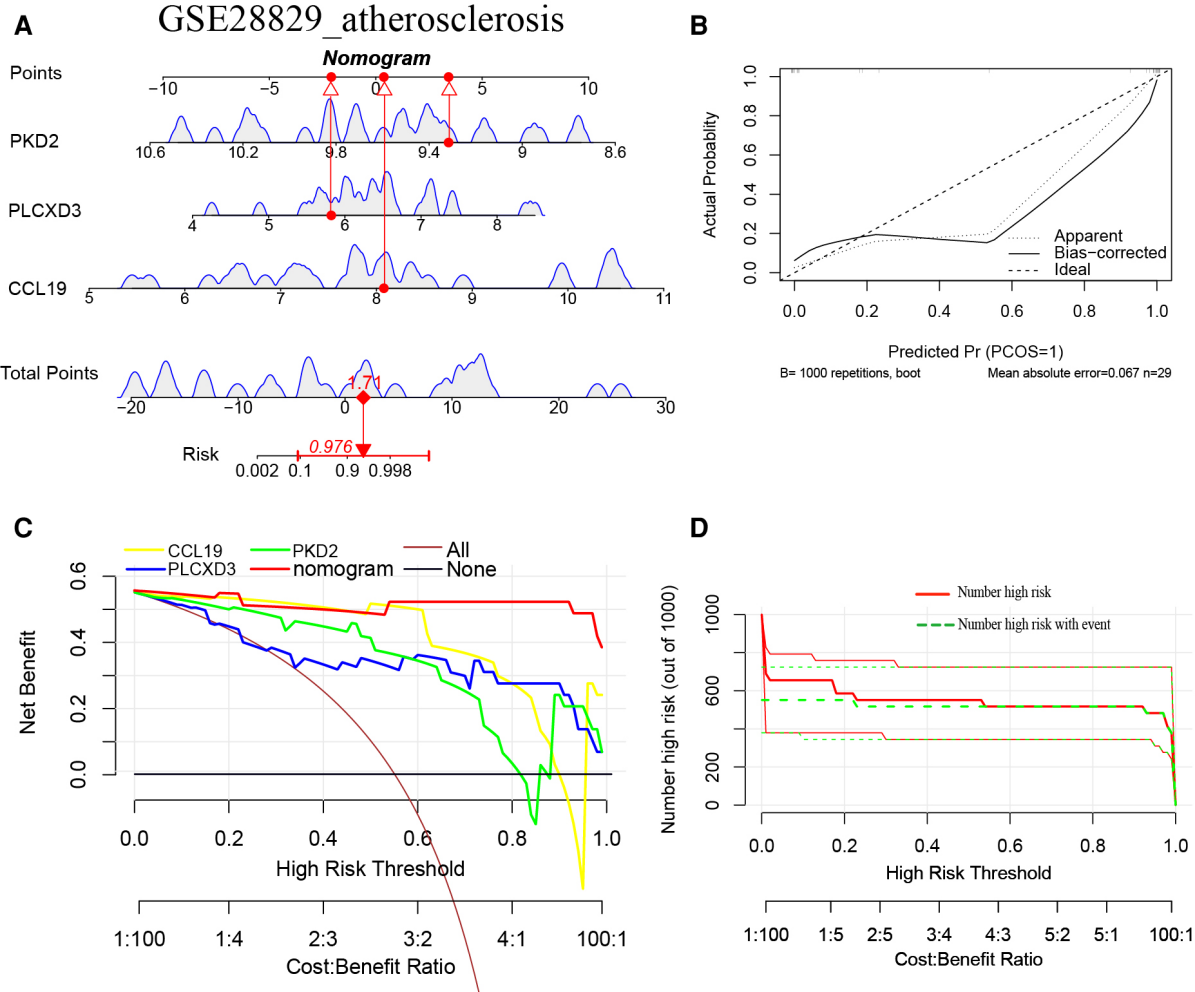
(**A**) The nomogram model predicting AS based on three common signature genes in GSE28829. The nomogram is used by summing all points identified on the scale for each variable. The total points projected on the bottom scales indicate the probabilities of AS; (**B**) the calibration curves for the nomogram with the mean absolute error = 0.067; (**C**) DCA of the nomogram model and each common signature gene (the “All” means diagnosis-all strategy; the “None” means diagnosis-none strategy) and the nomogram model had a higher net benefit at all practical risk thresholds (from 0 to 1.0); (**D**) the CIC of the nomogram model.

Furthermore, a nomogram model for NAFLD was constructed based on these three signature genes (PLCXD3, CCL19, and PKD2) *via* the R rms package (Figure 6A). Then, a calibration curve was used to evaluate the predictive power of the nomogram model. The calibration curve indicated that the error between the actual probability and predicted probability of advanced NAFLD is minimal in GSE49541, with a mean absolute error of 0.048. This result suggests that this nomogram model owns a high accuracy in predicting advanced NAFLD (Figure 6B). To estimate the clinical applicability of the prediction nomogram model, we execute DCA and CIC. As shown in Figure 6C that within all practical risk thresholds (from 0 to 1.0) and within the range that affects the prognosis of patients, the nomogram model always has an excellent overall net income. The “Number high risk” curve was close to the “Number high risk with event” curve at a high-risk threshold from 0.4 to 1, which indicated that the nomogram model owns extraordinary predictive power for advanced NAFLD (Figure 6D). Similarly, a nomogram model for advanced AS was established and evaluated in GSE28829. These signature genes also consistently have a favorable diagnostic efficiency for advanced AS (Figures 7A–D).

### Assessment of the nomogram model with training and verification sets

**Figure 8:**
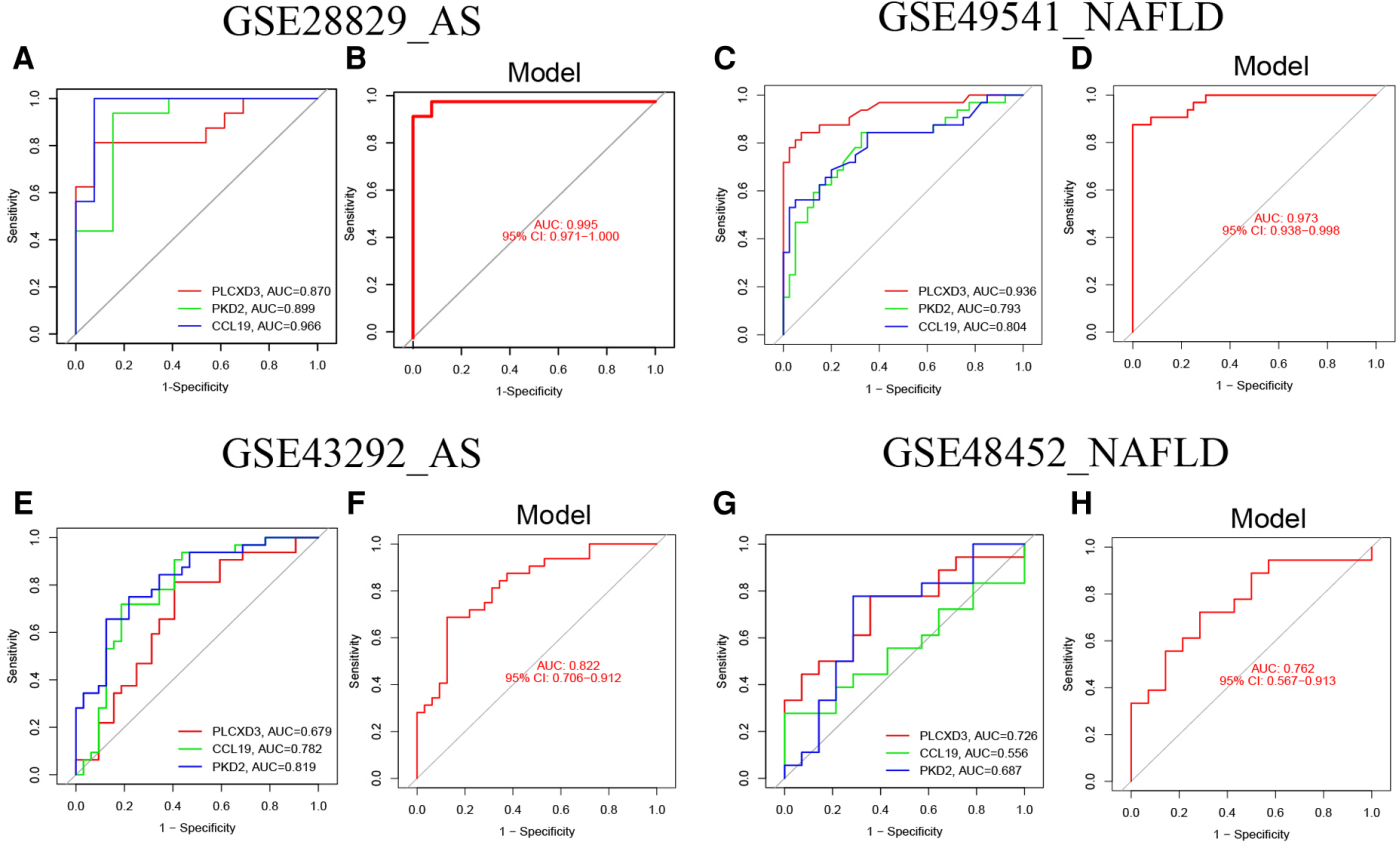
The validation of the nomogram models with ROC curves. (**A**) The ROC and AUC of each common signature gene in GSE28829; (**B**) the ROC and AUC of the nomogram model in GSE28829; (**C**) The ROC and AUC of each common signature gene in GES49541; (**D**) the ROC and AUC of nomogram model in GSE49541; (**E**) the ROC and AUC of each common signature gene in GSE43292; (**F**) the ROC and AUC of the nomogram model in GSE43292; (**G**) the ROC and AUC of each common signature gene in GES48452; (**H**) the ROC and AUC of the nomogram model in GSE48452.

ROC curves with AUC are shown to investigate the nomogram models’ diagnostic effectiveness for advanced AS and NAFLD using the identified three common signature genes. ROC curve analyses revealed that the AUC was 0.870 for PLCXD3, 0.899 for PKD2, and 0.966 for CCL19 in GSE28829 (Figure 8A). Additionally, the AUC was 0.995 [95% confidence interval (CI), 0.971–1.0] for the nomogram model by utilizing all common signature genes simultaneously in GSE28829 (Figure 8B). Homoplastically, the AUCs for each common signature gene were displayed in Figure 8C, and the nomogram model based on three common signature genes also owned a high accuracy (AUC = 0.973, 95% CI 0.938–0.998) (Figure 8D). Consistent with the training set, the AUCs of the nomogram models in two independent validation sets (GSE43292 of AS and GSE48452 of NAFLD) were 0.822 (95% CI 0.706–0.912) and 0.762 (95% CI 0.567–0.913), respectively (Figures 8E–H). These results suggested that these three common signature genes (PLCXD3, CCL19, and PKD2) and nomogram models can serve as effective diagnostic biomarkers for distinguishing advanced AS and NAFLD.

### Interaction network of common signature genes and their co-expression genes

**Figure 9:**
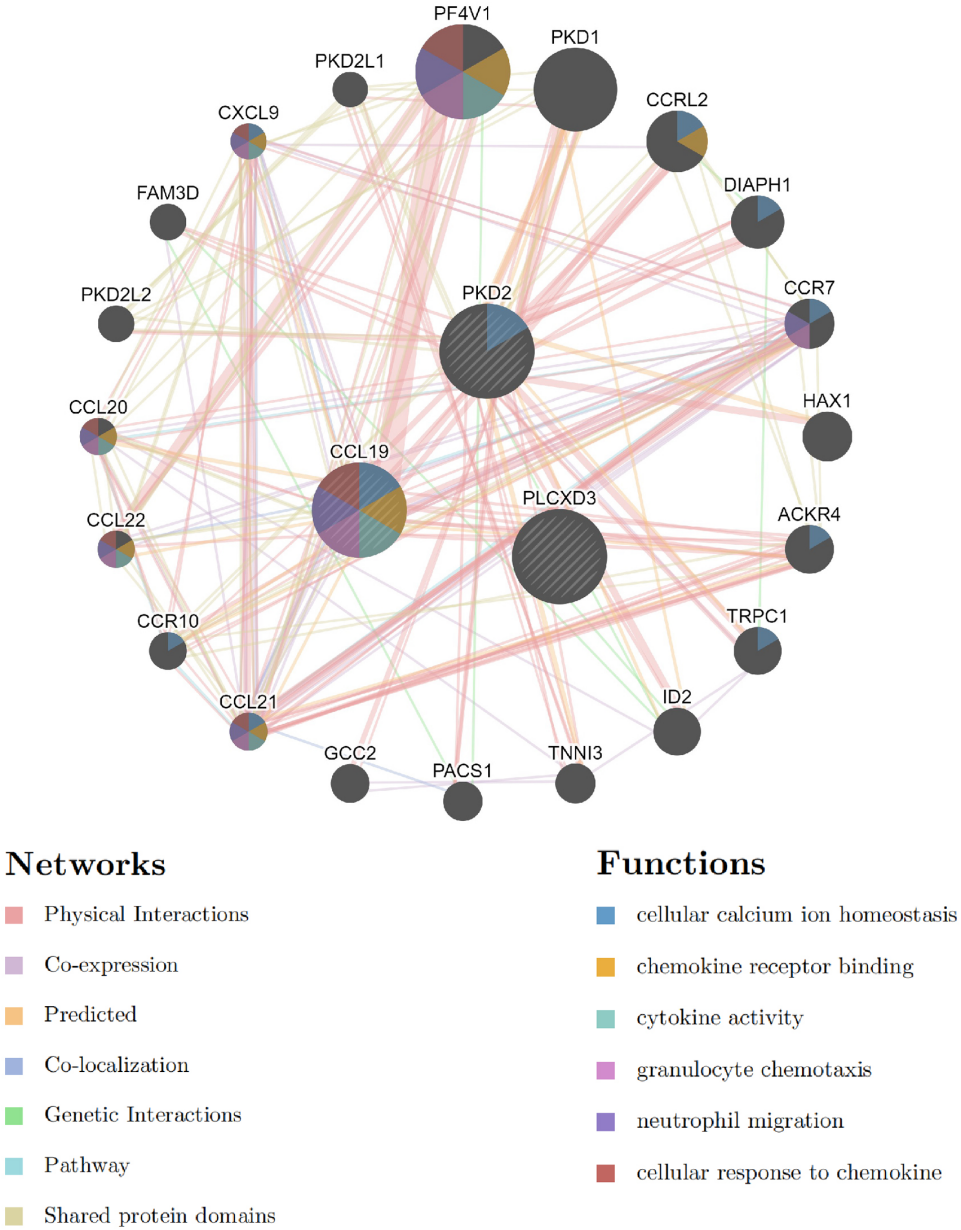
The common signature genes and their co-expression genes were analyzed *via* GeneMANIA.

We analyzed the interaction network and related functions of these common signature genes. These genes showed a complex interaction network with a physical interaction of 77.64%, co-expression of 8.01%, predicted of 5.37%, co-localization of 3.63%, genetic interactions of 2.87%, pathway of 1.88%, and shared protein domains of 0.60%. The biological functions of this interaction network were mainly involved in cellular calcium ion homeostasis, chemokine receptor binding, cytokine activity, granulocyte chemotaxis, neutrophil migration, and cellular response to chemokine (Figure 9 and Supplementary Material). CCL19 sits in a more central position in this interaction network by taking part in the most biological functions. These outcomes illustrated that inflammatory response and its associated pathways might participate jointly in the pathogenesis of these two diseases. Thus, the precisely common pathways need further investigation urgently.

### Identification of the co-regulated pathways *via* GSVA

**Figure 10:**
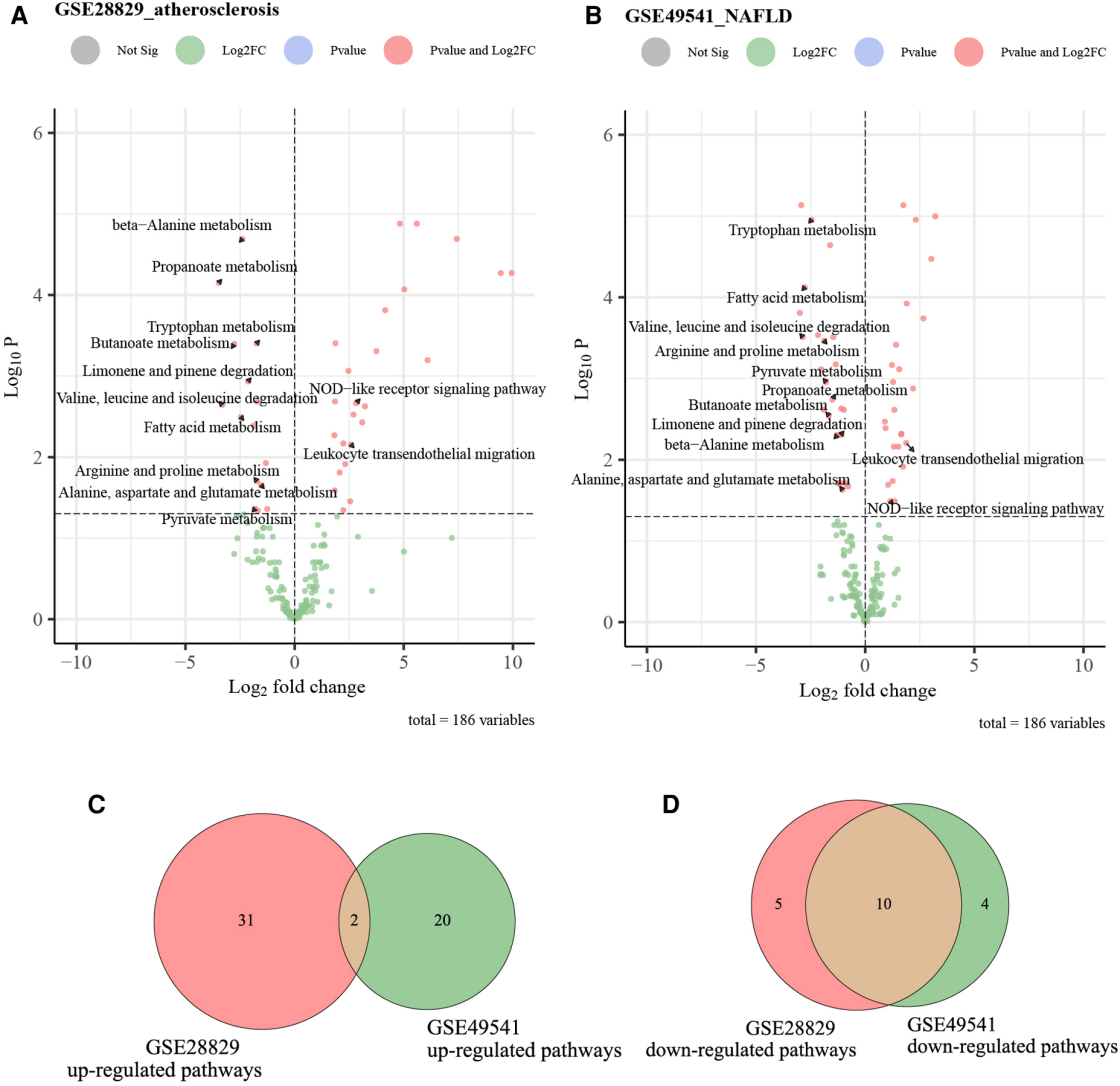
The volcano plots and Venn diagrams for identifying co-regulated pathways. (**A**) The volcano plot of up-regulated and down-regulated pathways in GSE28829; (**B**) the volcano plot of up-regulated and down-regulated pathways in GSE49541; (**C**) the Venn diagram of common up-regulated pathways between GSE28829 and GSE49541; (**D**) the Venn diagram of common down-regulated pathways between GSE28829 and GSE49541.

We utilized the GSVA analysis to explore the co-regulated pathways with consistent expression trends in GSE28829 and GSE49541, respectively. The results of GSVA analyses and Venn diagrams illustrated that the activities of two immune-related pathways (NOD-like receptor signaling pathway and leukocyte transendothelial migration) were simultaneously up-regulated in advanced AS and NAFLD groups (Figures 10A–C). Correspondingly, the activities of 10 metabolic-related pathways, especially fatty acid and amino acid metabolism, were simultaneously down-regulated (Figure 10D). These pathways will be candidates for further validation.

### Validation of the co-regulated pathways *via* single gene GSEA

**Figure 11:**
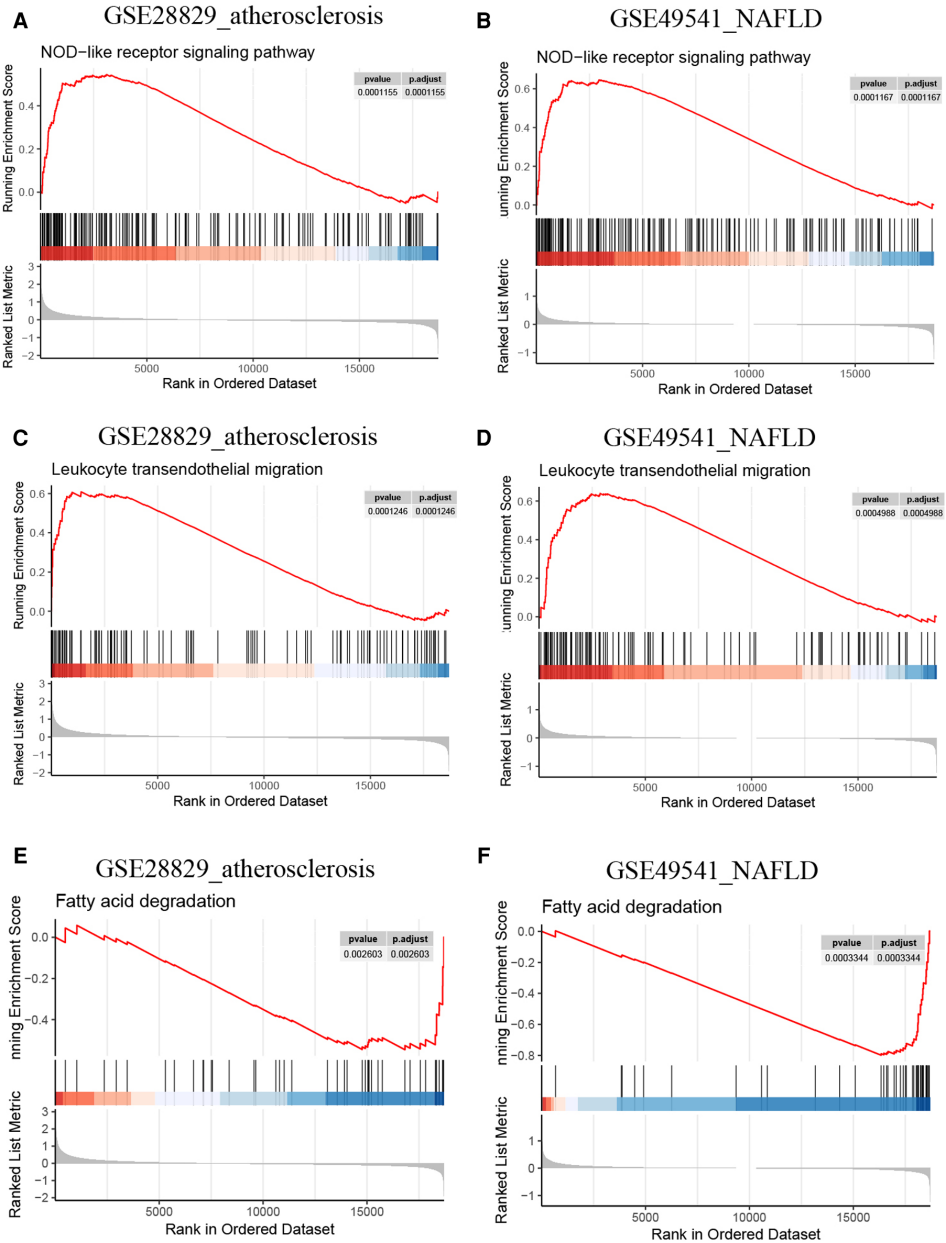
Single-gene GSEA of CCL19. (**A**) The NOD-like receptor signaling pathway from single-gene GSEA in GSE28829; (**B**) the NOD-like receptor signaling pathway from single-gene GSEA in GSE49541; (**C**) the leukocyte transendothelial migration from single-gene GSEA in GSE28829; (**D**) the leukocyte transendothelial migration from single-gene GSEA in GSE49541; (**E**) the fatty acid degradation from single-gene GSEA in GSE28829; (**F**) the fatty acid degradation from single-gene GSEA in GSE49541.

Since PLCXD3, CCL19, and PKD2 might be pivotal in the progression of advanced AS (GES28829) and NAFLD (GSE49541), we selected these genes separately for further single-gene GSEA to confirm the findings of GSVA. The conclusions were primarily consistent with the previous results. Both the leukocyte transendothelial migration and NOD-like receptor signaling pathway own a higher activity in the high CCL19 group (Figures 11A–D). Inversely, the up-regulation of CCL19 was closely associated with the down-regulated fatty acid degradation (Figures 11E,F). Interestingly, several classic immune-related pathways, such as the toll-like receptor signaling pathway, TNF signaling pathway, and Th1 and Th2 cell differentiation, also have a high activity due to the perturbation of CCL19 (Supplementary Figures S1A–F). Similarly, the single-gene GSEA outcomes of PLCXD3 and PKD2 were mainly consistent with the above (Supplementary Material).

### Immune infiltration analysis

**Figure 12:**
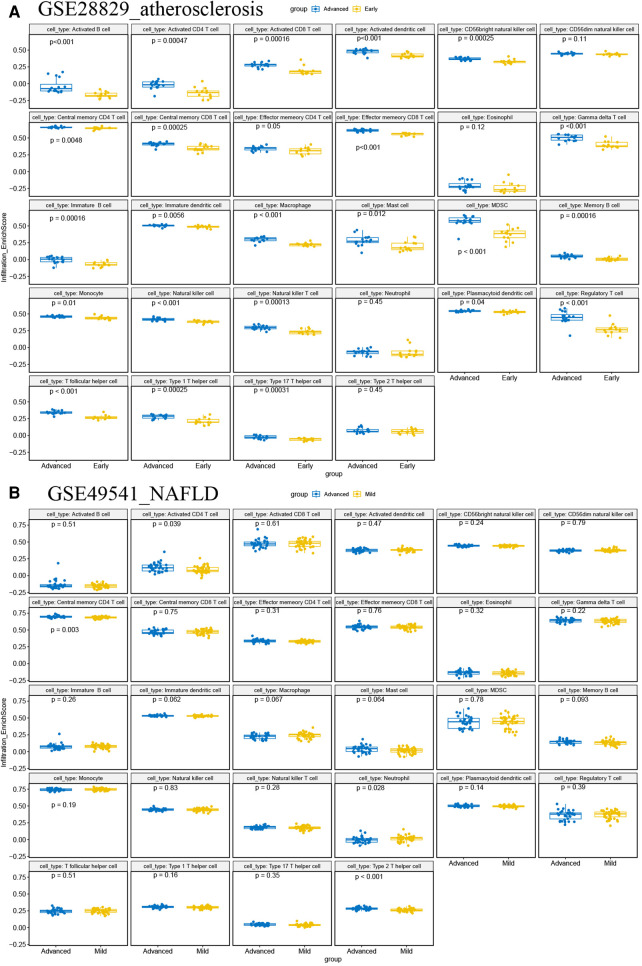
ssGSEA for immune infiltration. (**A**) The boxplots of 28 immunocyte subtypes between advanced and early AS in GSE28829; (**B**) the boxplots of 28 immunocyte subtypes between advanced and mild NAFLD in GSE49541.

It is reported that AS and NAFLD are inflammatory-related diseases characterized by the infiltration of immune cells into plaques and hepatic lobules. Our study also revealed that multiple immune-related pathways might promote the progression of AS and NAFLD, as previously described. Then two disparate algorithms were applied to identify the heterogeneous infiltration of immune cells in AS and NAFLD. Firstly, we used the ssGSEA to identify immune cell subtypes that are differentially represented in the advanced and early AS, while the immune-related genes set of 28 immune cell subtypes was derived from 37 studies of microarray data (27). As shown in Figure 12A, almost all of these subtypes of immune cells (especially the T-lymphocyte, macrophage, dendritic cell, and mast cells) were excessively enriched in advanced AS plaques compared to the early ones, which indicated that the excessive activation of immune cells has a regulatory effect on promoting AS. In the advanced NAFLD tissues, activated CD4 T cells, central memory CD4 T cells, and type 2 T helper cells were significantly more than mild NAFLD (GSE49541) (Figure 12B).

**Figure 13:**
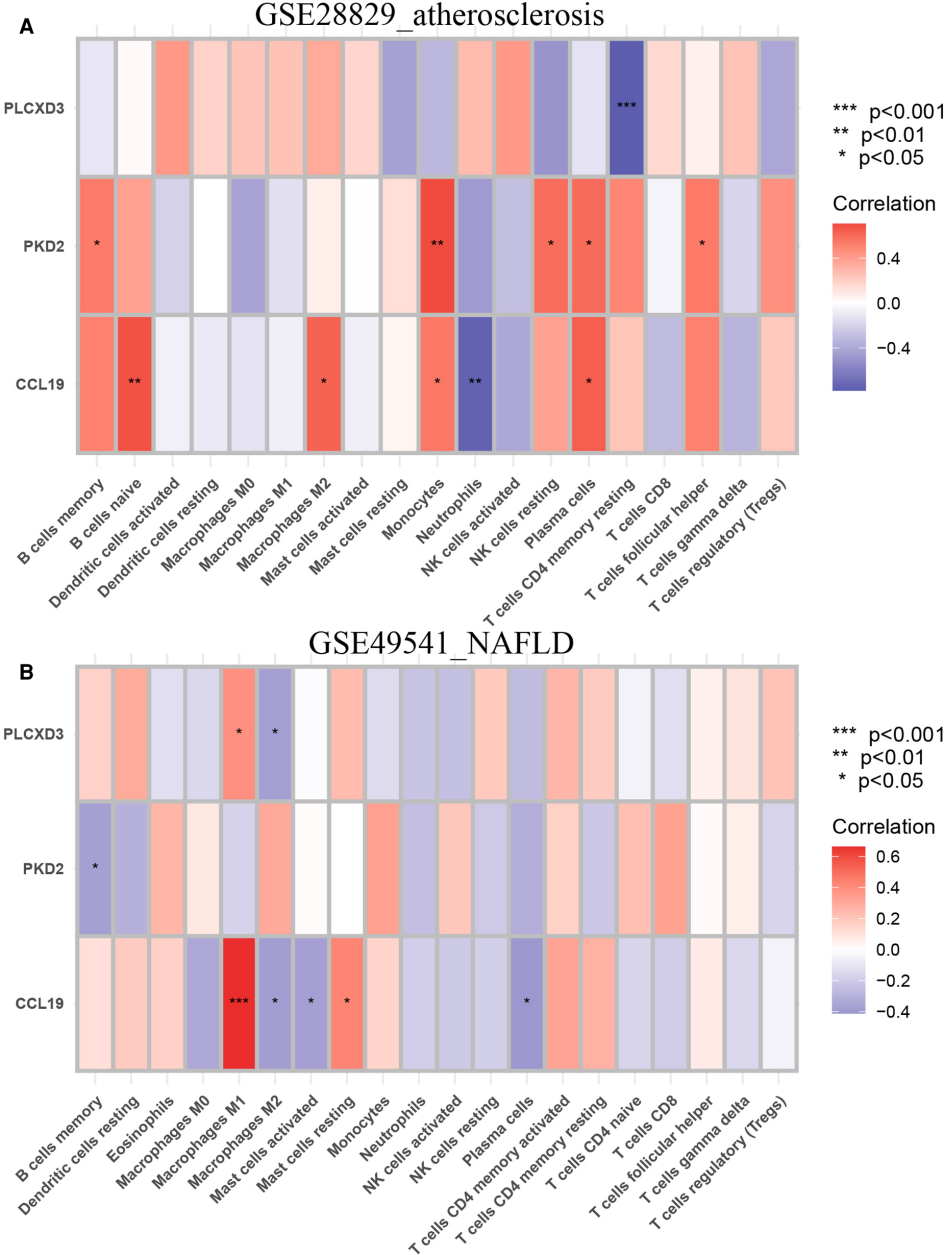
Immune infiltration analysis of the CIBERSORT algorithm. (**A**) The heatmap of the correlation coefficient between each common signature gene and each immunocyte subtype in GSE28829; (**B**) the heatmap of the correlation coefficient between each common signature gene and each immunocyte subtype in GSE49541.

Next, the correlations between each subpopulation of immune cells and each signature gene were performed based on Pearson's correlation coefficient *via* the CIBERSORT algorithm, as the heatmaps show that each signature gene was significantly associated with one or more subtypes of immune cells, especially the CCL19 belonging to chemokines (Figures 13A,B). The violin plots of the expression levels of 22 immunocyte subtypes in GSE28829 and GSE49541 are displayed in Supplementary Figures S2A,B. Summarily, the inflammation process plays a critical role in advanced AS and NAFLD, and they share some common characteristics in this issue.

## Discussion

Inflammation, an innate and integrated response to pathogens, irritants, immune mediator, and chemicals, also lead to tissue damage if immoderate or unbalanced. NAFLD and AS are inflammation-related diseases jointly mediated by metabolic and immune factors. It has well known that various cytokines, excessive inflammation cascades, inappropriate inflammation processes, and activated immune cell infiltration profoundly disturb endarterium physiology and promote atherosclerosis (28). Many studies indicated that inflammation exacerbates the metabolic disorders of NAFLD, and metabolic inflammation plays a crucial role in the pathogenesis and progression of NAFLD to advanced liver disease. Moreover, the long-term prognosis of NAFLD is closely correlated with the dysfunction of metabolic-inflammatory signaling (29). Many cytokines and signal pathways, such as IL-1β and PI3K/Akt/mTOR pathway, are confirmed to drive the dysregulation of hepatic metabolism and inflammation (30). It has been proposed that NAFLD is closely associated with chronic inflammation and insulin resistance, and hyperinsulinemia promotes hepatic lipogenesis, further exacerbating insulin resistance (31, 32). The development of hepatic insulin resistance can trigger lipogenesis, contributing to the progression of NAFLD, dyslipidemia, and AS (33). Along the line, we hypothesize that NAFLD and AS might share overlapping pathogenic DEGs and pathways. However, the integrated studies that focus on comprehensively analyzing the molecular mechanism, pathways, and immune infiltration characteristics of advanced AS and NAFLD co-pathogenesis are still limited. Thus, we perform this study from the transcriptome perspective through the public data.

As previously described, we analyzed the advanced NAFLD and AS transcriptomic data and finally identified three common signature genes (PLCXD3, CCL19, and PKD2). Then the nomogram models with extraordinary predictive efficacy were established and confirmed for advanced NAFLD and AS with the calibration curve, DCA, and CIC. The ROC curves of training sets were applied to evaluate the availability of the nomogram models with the AUC = 0.995 (95% CI 0.971–1.0) for advanced AS and AUC = 0.973 (95% CI 0.938–0.998) for advanced NAFLD. Furthermore, the AUC of the verification sets had a similar trend. These outcomes indicated that PLCXD3, CCL19, and PKD2 might play an essential role in developing NAFLD and AS, contributing to effectively diagnosing these two diseases.

We also identified the co-regulated pathways between advanced NADLAD and AS *via* GSVA. Two immune-related pathways (NOD-like receptor signaling pathway and leukocyte trans endothelial migration) are simultaneously upregulated in the advanced AS and NAFLD groups. A total of 10 metabolic-related pathways were simultaneously downregulated, including fatty acid metabolism. These results illustrated that these two diseases own common inflammatory pathways and metabolic disorders. NLRs involve in the inflammatory response and promote programmed cell death. Much attention has been paid to its critical role in the pathogenesis of metabolic diseases, such as NAFLD, type 2 diabetes mellitus (T2DM), hypertension, and AS (34). Inflammasomes are crucial regulators of innate immunity, contributing to atherogenesis by being activated within macrophages and artery walls. It is suggested that the NLRs protein inflammasome and its stimulation of innate immunity is a strong promotor of AS (35). IL-1β and IL-18, two atherogenic cytokines, are matured in NLRPs inflammasomes. Further research revealed that NLRP3 inflammasome is expressed in atherosclerotic plaque (36). Current evidence suggests that innate immunity is an essential accelerator in NAFLD progression, and NLRs drive NASH (37). Furthermore, cardiolipin can activate the up-regulated NLRP3 inflammasome and promote NASH pathogenesis (38). In summary, the NLRs signaling pathway and NLRPs inflammasomes were co-upregulated in advanced NAFLD and AS, providing a novel potential treatment for these two diseases.

CCL19 is a member of chemokine ligands, taking part in inflammatory responses and normal lymphocyte recirculation and homing (39). It specifically binds to chemokine receptor CCR7 and shows potent chemotactic activity for regulating T cells activation (40). According to GO analysis and gene-interaction network, CCL19 was crucial in positively regulating cytokine production, NIK/NF-KB signaling, cytokine activity, chemokine receptor binding, and inflammatory cell migration. It was detected that CCL19 was significantly and positively associated with inflammatory signaling pathways such as toll-like receptor 4 (TLR4)/NF-KB and proinflammatory factors, including IL-6 and TNF-α in NAFLD patients. Additionally, metformin can significantly suppress the high expression of CCL19 and improve NAFLD, demonstrating that inhibition of CCL19 may be an effective treatment for NAFLD (41). The CCL19/CCR7 pathway promotes the progression of high-fat-induced IR and obesity (42), and these issues are well-known as risk factors for accelerating the pathogenesis of NAFLD and AS. CCL19/CCL21-CCR7 is closely correlated with high coronary artery disease risk and is considered a novel homeostatic chemokine system that promotes atherogenesis by modulating monocyte adhesion and migration (43). Salem MK reported that CCL19 is significantly over-expressed in unstable carotid atherosclerotic plaques (44). Inversely, the deletion of CCR7 in mouse AS contributes to the reduced atherosclerotic plaque content through regulating T cells and antigen-presenting dendritic cells (DCs) (45).

To our knowledge, this is the first study demonstrating that CCL19 was significantly and simultaneously co-upregulated in advanced NAFLD and AS. As the previous references and our findings suggested, CCL19 might mediate the pathogenesis of advanced NAFLD and AS by regulating the immune-related pathways and inflammatory cell migration. Then we applied CCL19-specific single-gene GSEA to explore the critical mechanisms within these two diseases. Exhilaratingly, the up-expression of CCL19 is positively associated with the activation of NOD-like receptors signaling pathway, leukocyte transendothelial migration, toll-like receptor signaling pathway, TNF signaling pathway, and Th1 and Th2 cell differentiation, which corresponds with the literature and supports our hypothesis. Moreover, the high expression of CCL19 was significantly associated with reduced activity of fatty acid degradation, which might lead to dyslipidemia and trigger the advance of NAFLD and AS. Therefore, targeting CCL19 may provide a therapeutic method for decelerating the progression of advanced NAFLD and AS.

However, few studies that focus on the correlation between PLCXD3, PKD2, and these two relevant diseases, and direct evidence is also lacking. A Previous study has illustrated that down-regulated expression of PLCXD3, a member of the phosphoinositide-specific phospholipases (PI-PLC) family, suppressed insulin secretion due to the disruption of the necessary insulin signaling pathways and insulin biosynthesis genes in islet β-cells (46). Taneera Jalal revealed and confirmed PLCXD3 as a potential regulator of pancreatic islet function (47). An Emiratis population-based cross-sectional study indicated that genetic variants of the PLCXD3 are correlated with lower HDL-cholesterol and MS risk (48). According to GO analysis, as previously described, PLCXD3 was closely related to the lipid catabolic process. These outcomes were consistent with the literature review, which suggested that up-regulated PLCXD3 might contribute to the progression of NAFLD and AS due to its involvement in hyperinsulinemia and dyslipidemia. We found that PKD2, also called transient receptor potential polycystin-2 (TRPP2), plays a vital role in calcium ion transport and cellular calcium ion homeostasis based on enrichment analysis and gene interaction network. It is a Ca^2+^ channel located on the membrane of the cell surface and endoplasmic reticulum (ER), closely associated with various cellular functions. Hasan Raquibul reported that SUMO1 modification of PKD2 channels regulates arterial contractility (49). Current knowledge regards PKD2 may regulate the functions of endothelial cells, vascular smooth muscle cells, and blood pressure (50). The mutation of the PKD2 leads to a systemic disorder of vasculature (51). The dysfunction of calcium homeostasis in hepatic mitochondria can lead to excess lipid absorption and metabolism disorders, which have been regarded as a potential mechanism to accelerate NAFLD progression (52). Based on our bioinformatics analyses, we found that PLCXD3 and PKD2 might provide a novel perspective to understand the disease progression of advanced NAFLD and AS, and these results might serve as a theoretical basis for further experimental studies in this direction.

Finally, we were concerned with exploring the analogical feature of immune cell infiltration among advanced NAFLD and AS. Various immune cells, such as macrophages, DCs, and T cells, participate in and drive lipid deposition and peroxidation, ultimately promoting atherogenesis (53–55). The compositions of circulating and intrahepatic immune cells were also polymorphic in patients with fatty liver and steatohepatitis (56). Interestingly, we found that activated CD4 T cells and central memory CD4 T cells were significantly excessive infiltration in advanced NAFLD and AS, which had a similar trend. In the hyperlipidemic μMT^−/−^ ApoE^−/−^ mice model, wildtype B cells can accelerate atherogenesis and increase CD4 T cells in plaques, including memory and activated CD4 T cells. These outcomes indicated that targeting the interaction of B cells and CD4 T cells may be a therapeutic strategy to restrict AS progression (57).

Furthermore, novel evidence tends to confirm that the dysbiosis of NASH may drive the migration of CD4 T cells from intestinal and mesenteric lymph-nodal into the liver (58). Other studies also found rather increased numbers of intrahepatic CD4 T cells in murine models of NASH, such as western and high-fat diets (59). Central and effector memory CD4 T cells play an active role in promoting and sustaining liver high-fat-diet related murine model of NASH, accompanied by marked up-regulation of pro-inflammatory cytokines IL-17A and IFN-γ. Then depletion of CD4 T cells leads to abrogate the intrahepatic immune infiltration, inflammation, and fibrosis (60). Using an experimental transgenic murine model, Nabil D showed that the hepatocyte damage triggers a high releasing amount of IL-17. Some studies revealed that IL-17 activates lipolysis of the white adipose tissue and actively promotes the progression of hepatic steatosis and NASH. Additionally, a high concentration of circulating IL-17 was reported in NASH patients (59). Similarly, previous studies revealed that IL17 contributes to vascular and systemic inflammation in experimental AS (61), and the frequencies of IL17+ CD4 T cells significantly increase in the severe coronary AS group (62). Thus CD4 T cells, combined with their relevant pro-inflammatory cytokines, might contribute to NAFLD and AS co-morbidities through immune and inflammatory pathways. The polymorphism and variability of immune cell infiltration is a great challenge and expect to become a novel research topic.

Because of the substantial metabolic similarity between NAFLD and AS, many researchers have focused on this field. However, studies have yet to explore the common molecular mechanism and pathways between these two relevant diseases *via* advanced bioinformatics methods. Unlike previous studies, our study simultaneously pays more attention to exploring common signature genes, related co-regulated pathways, and immune characteristics. Due to the high comorbidity rate between NAFLD and AS, we have applied an integrated method based on bioinformatics and machine learning algorithms, which have been proven to be credible in various diseases, to identify the common signature genes and pathways for the first time (63). These findings may further clarify the sharing mechanism of advanced NAFLD and AS. Our study also had some limitations. Significantly, this is a retrospective study that requires further experiments and clinical data to corroborate our outcomes in the future. Fortunately, the diagnostic models base on three signature genes were also efficient in external validation sets, which would partially enhance the credibility of our results. Moreover, it should be emphasized that the direct diagnosis of advanced NAFLD and AS could not base on these common signature genes and pathways, and invasive biopsy and pathological confirmation at histology are needed. However, our findings might provide new insights and biomarkers for the common molecular mechanisms of advanced NAFLD and AS. Because of the close correlation between clinical factors and these two diseases, integrating these signature genes with other clinical diagnostic models and targeting them might also will be considerable and valuable.

## Conclusion

We identified three common signature genes (PLCXD3, CCL19, and PKD2), co-regulated pathways, and shared immune features of advanced NAFLD and AS, and then established effective diagnosis models. We found that these two related diseases shared many common pathogenic mechanisms. This study might provide novel insights into the molecular mechanism of advanced NAFLD complicated with AS from the multi-dimensional perspective of genetics, signaling pathways, and immune infiltration.

## Data Availability

Publicly available datasets were analyzed in this study. This data can be found here: https://www.ncbi.nlm.nih.gov/geo/query/acc.cgi?acc=GSE28829; https://www.ncbi.nlm.nih.gov/geo/query/acc.cgi?acc=GSE49541; https://www.ncbi.nlm.nih.gov/geo/query/acc.cgi?acc=GSE43292; https://www.ncbi.nlm.nih.gov/geo/query/acc.cgi?acc=GSE48452.
