# Supplementary material for: Identification of common signature genes and pathways underlying the pathogenesis association between nonalcoholic fatty liver disease and atherosclerosis
Source: Front Cardiovasc Med. 2023 Mar 30;10:1142296. doi: 10.3389/fcvm.2023.1142296 (PMC10098172; doi:10.3389/fcvm.2023.1142296)
Supplement: Supplementary file 2 [file Datasheet1.doc]

**Links to raw data and worksheets**

https://www.jianguoyun.com/p/Dem1My4Q-aurCxjI6_IEIAA
