# Supplementary figures and images for: Identification of common signature genes and pathways underlying the pathogenesis association between nonalcoholic fatty liver disease and atherosclerosis
Source: Front Cardiovasc Med. 2023 Mar 30;10:1142296. doi: 10.3389/fcvm.2023.1142296 (PMC10098172; doi:10.3389/fcvm.2023.1142296)

A

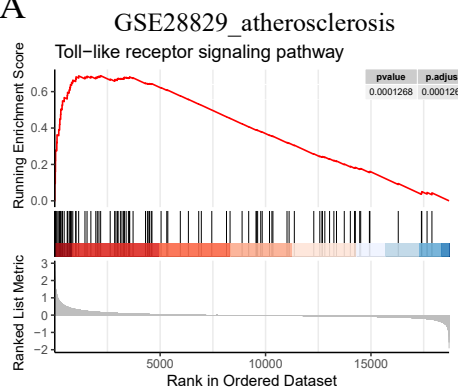

B

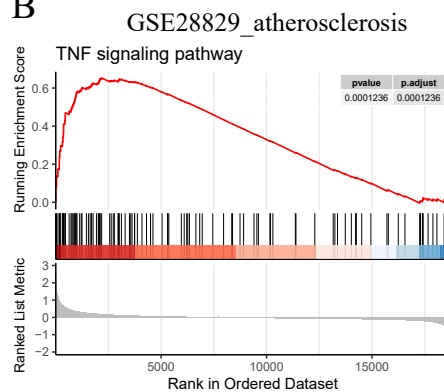

C

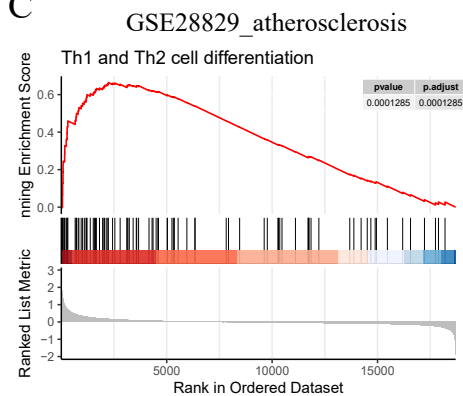

D

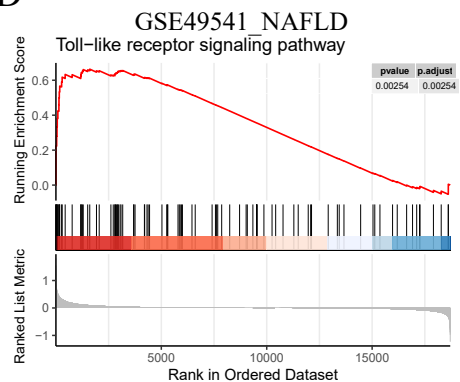

E

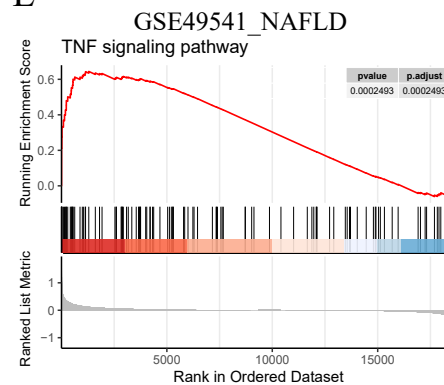

F

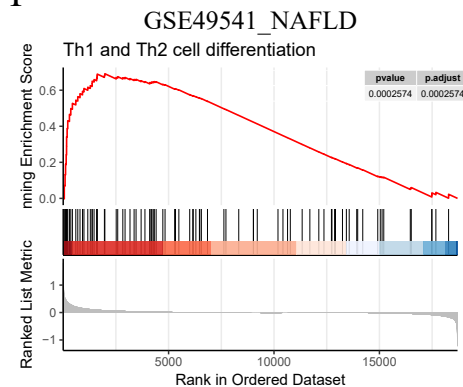

Supplement: Supplementary file 3 [file Image1.pdf]

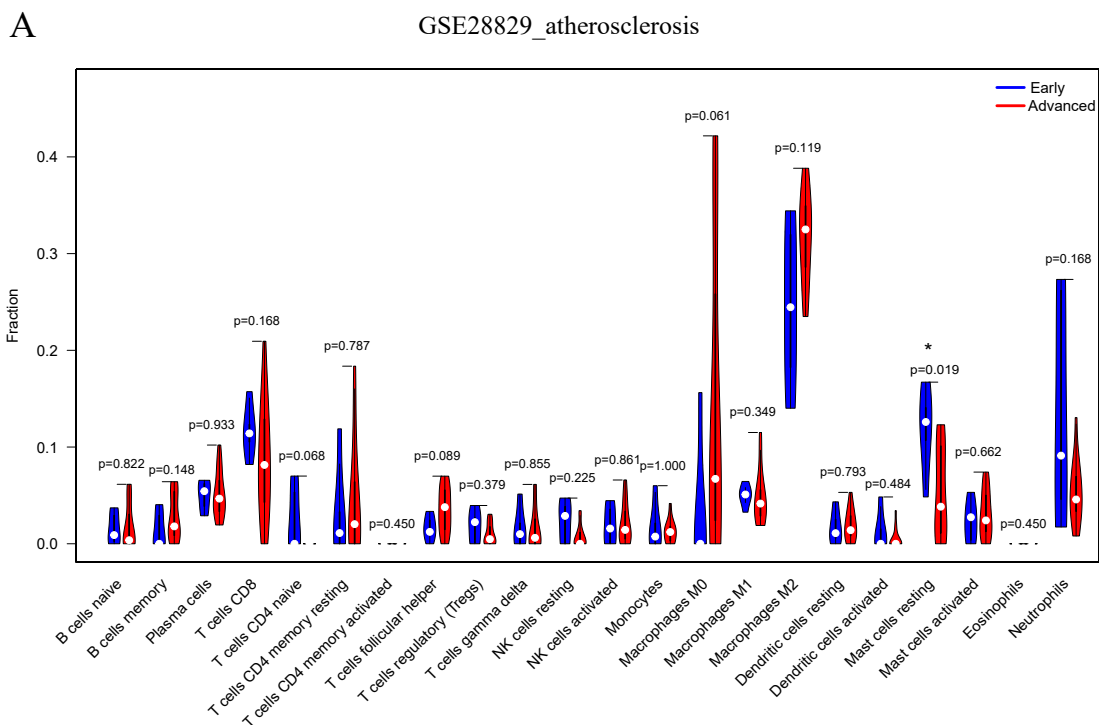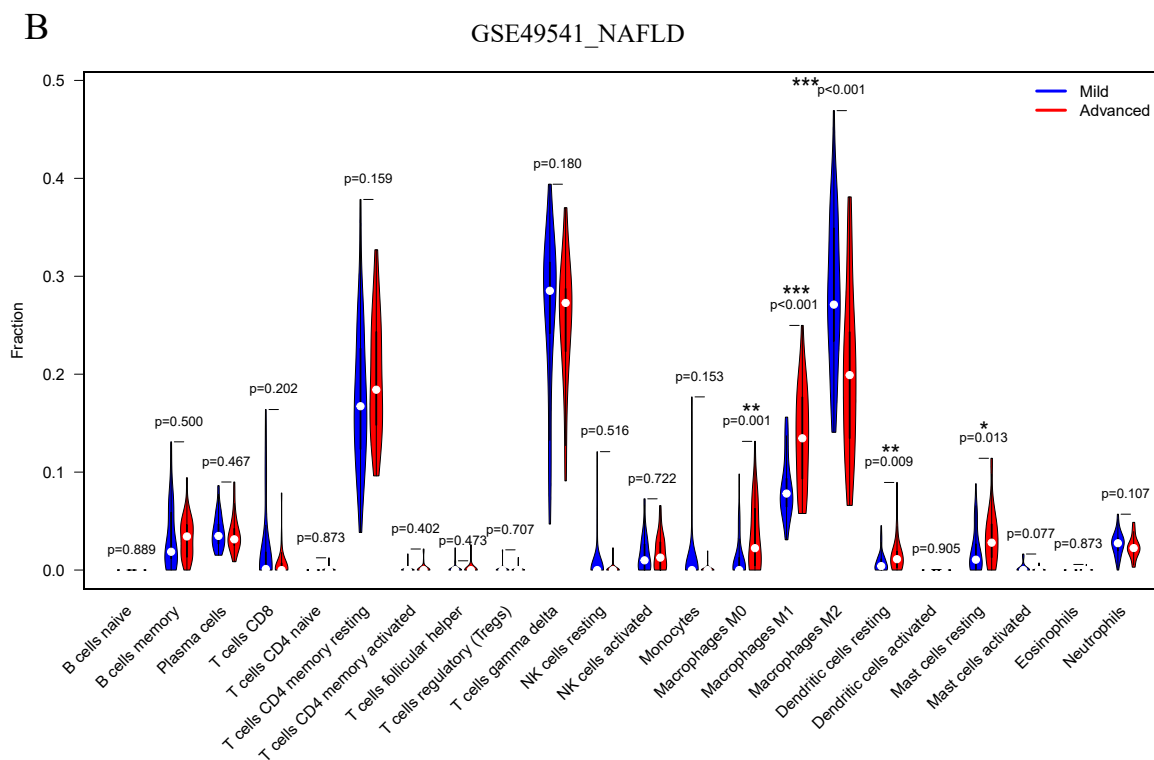

Supplement: Supplementary file 4 [file Image2.pdf]
